# Alzheimer's Disease Puzzle: Delving into Pathogenesis Hypotheses

**DOI:** 10.14336/AD.2023.0608

**Published:** 2024-02-01

**Authors:** Mohammad Nasb, Weichu Tao, Ning Chen

**Affiliations:** Tianjiu Research and Development Center for Exercise Nutrition and Foods, Hubei Key Laboratory of Exercise Training and Monitoring, College of Sports Medicine, Wuhan Sports University, Wuhan 430079, China

**Keywords:** Alzheimer's disease, amyloid cascade hypothesis, Tau hyperphosporylation hypothesis, neuroinflammation hypothesis, mitochondrial dysfunction hypothesis

## Abstract

Alzheimer's disease (AD) is a prevalent neurodegenerative disease characterized by both amnestic and non-amnestic clinical manifestations. It accounts for approximately 60-70% of all dementia cases worldwide. With the increasing number of AD patients, elucidating underlying mechanisms and developing corresponding interventional strategies are necessary. Hypotheses about AD such as amyloid cascade, Tau hyper-phosphorylation, neuroinflammation, oxidative stress, mitochondrial dysfunction, cholinergic, and vascular hypotheses are not mutually exclusive, and all of them play a certain role in the development of AD. The amyloid cascade hypothesis is currently the most widely studied; however, other hypotheses are also gaining support. This article summarizes the recent evidence regarding major pathological hypotheses of AD and their potential interplay, as well as the strengths and weaknesses of each hypothesis and their implications for the development of effective treatments. This could stimulate further studies and promote the development of more effective therapeutic strategies for AD.

## 1. Introduction

Alzheimer's disease (AD) is a multifactorial neurodegenerative disease characterized by memory loss, impaired cognitive capacity, and abnormal behavior [1]. AD is one of the most significant inducers of dementia in geriatric patients, and its prevalence is increasing as the rapid population aging [2]. The onset of AD typically occurs in individuals over the age of 65, but early-onset AD can also be observed in individuals as young as 30 years old [3]. AD development can be experienced over a long period of time, with brain defense maintaining stability for roughly 20 years before there is cognitive decline at moderate stages of AD [4, 5]. The cellular defense phase is characterized as the biological equivalent of pre-clinical AD [6]. Histopathologically, AD is characterized by two features, including extracellular amyloid-beta (Aβ) aggregates, and intracellular phosphorylated Tau deposition, which stimulate the formation of neurofibrillary tangles (NFTs) in the subcortical gray matter and cerebral cortex [7]. AD can be classified as sporadic or familial AD. The sporadic type constitutes up to 95% of AD cases and is characterized as late-onset [8]. AD is distinctly different than normal aging as shown in Table 1.

**Figure 1.**
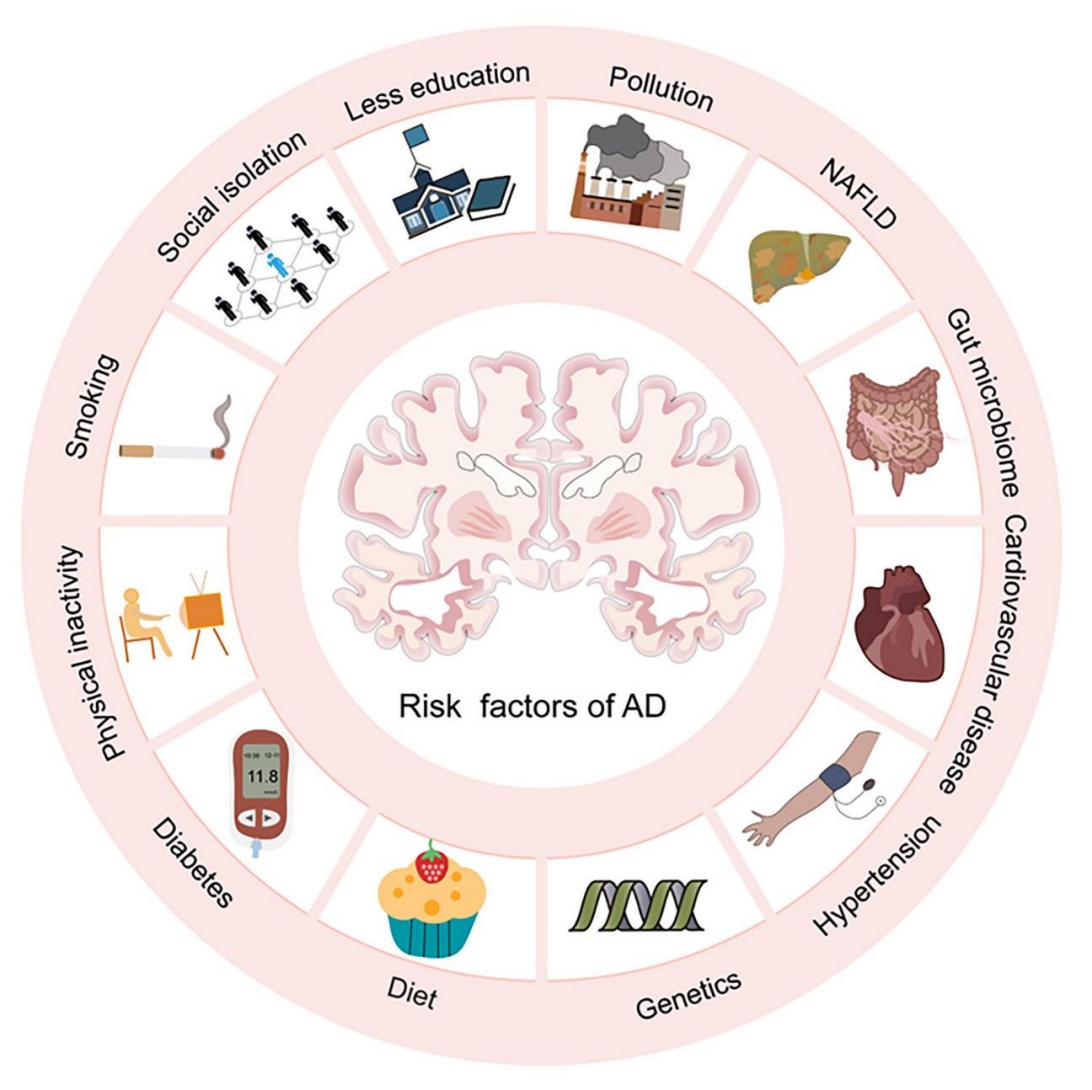
The major identified risk factors of AD. NAFLD: non-alcoholic fatty liver disease.

Currently, there is no cure for AD, but several treatments can slow down its progression and relieve symptoms. Risk factors for AD include age, genetics, familial history of AD, and health status, such as the presence of diabetes, high blood pressure, and high cholesterol. Many studies are being conducted to identify additional risk factors such as stress, lifestyle, and environmental factors [9] (Fig. 1). Additionally, ongoing studies on potential treatments, such as immunotherapy, stem cell therapy, and gene therapy, have demonstrated promising results in animal model studies.

## 2. Amyloid cascade hypothesis

**Figure 2.**
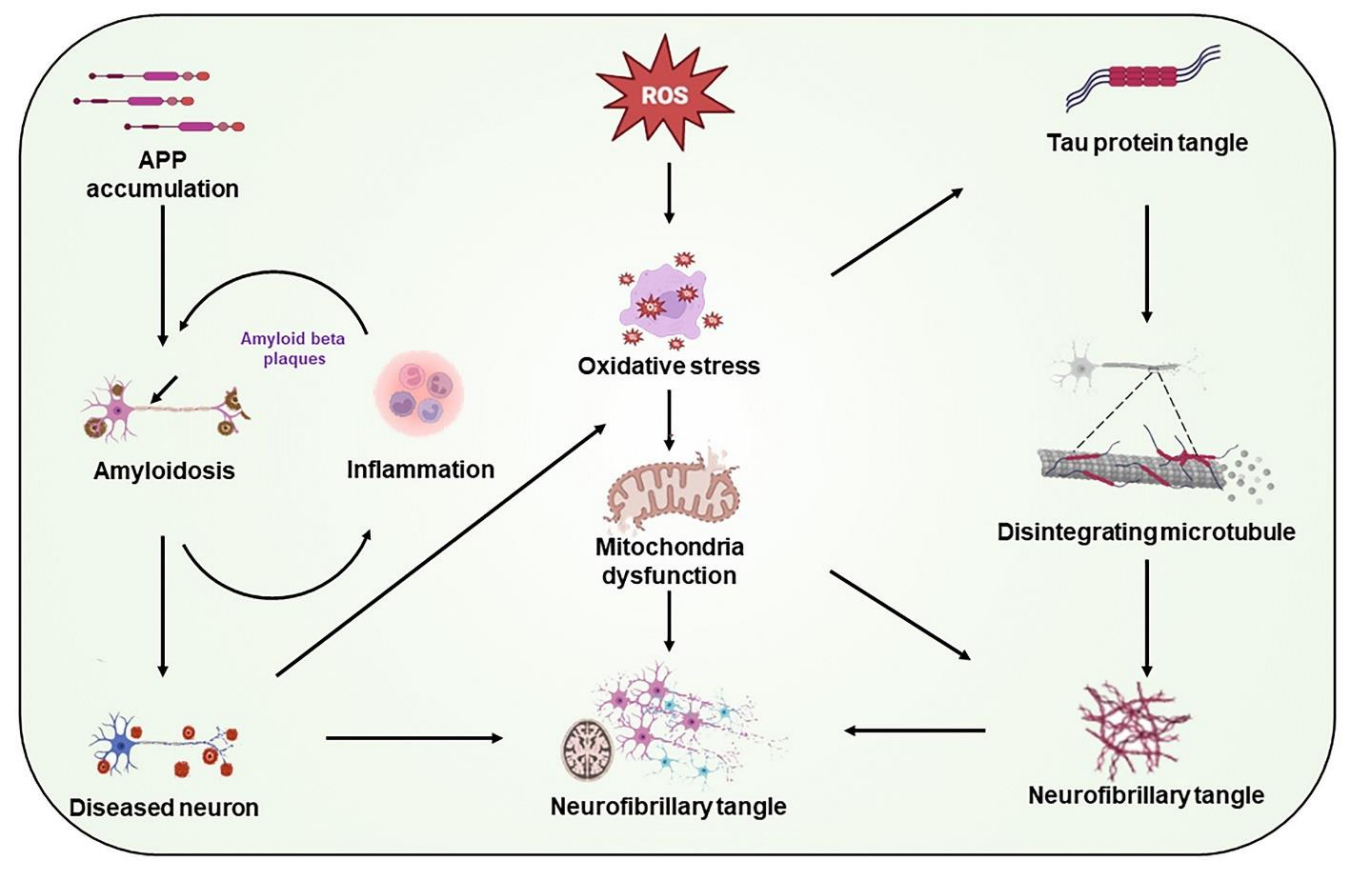
The proposed neurobiological mechanisms involved in the pathogenesis of AD.

The amyloid cascade hypothesis proposes the accumulation of Aβ plaques in the brain as the leading cause of AD [10]. This hypothesis suggests that normal clearance mechanisms for Aβ in AD conditions are disrupted, thus leading to the accumulation of Aβ and the formation of Aβ plaques deposits (Fig. 2) [11].

Aβ is a peptide produced by the proteolytic processing of amyloid precursor protein (APP) by beta-secretase (β-secretase) or gamma-secretase (γ-secretase) in brain tissues of individuals with AD [12]. There are several different forms of Aβ peptides, and they are distinguished by their lengths. The most common forms are Aβ40 and Aβ42. Aβ42 is considered more toxic than Aβ40, and it is more prone to the aggregation and formation of Aβ plaques in brain tissues [13]. Aβ peptides are present in β-sheet conformation aggregates and polymerized plaques, where they generate in structurally different forms, such as polymorphic and fibrillar oligomers [14].

The accumulation of Aβ protein is the beginning of a series of events that lead to a cascade of actions causing the death of nerve cells and damage to brain tissue, finally resulting in the symptoms of AD. According to the amyloid cascade hypothesis, the buildup of Aβ triggers a cascade of neurodegenerative processes, such as inflammation, oxidative stress, and the accumulation of Tau protein, ultimately leading to the symptoms of dementia. Studies on the amyloid cascade hypothesis have stimulated the development of several potential treatment strategies for AD, such as immunotherapy with the aim of clearing Aβ plaques from the brain, and β-site amyloid precursor protein cleaving enzyme 1 (BACE1) inhibitors for targeting the BACE enzymes responsible for Aβ production [15]. Although the amyloid cascade hypothesis is the most studied hypothesis, it does not fully explain the complex etiology of AD, due to the involvement of other factors in the development of this disease.

**Table 1:** Comparison of AD and normal aging.

Category	Alzheimer's disease	Normal aging
Symptom	Memory loss, difficulty with language and self-care, disorientation, personality changes, mood swings	Occasional memory lapse, slower processing speed, decreased ability to multitask, less efficient cognitive processing
Diagnosis	Requires comprehensive neurological and cognitive testing, medical history review, and imaging tests (MRI, or CT scan)	Diagnosis is typically not necessary unless there are significant changes in cognitive function or other symptoms
Treatment	Medications to manage symptoms, cognitive therapy, lifestyle changes (exercise, healthy diet), supportive care	Lifestyle changes (exercise, healthy diet), supportive care
Prognosis	Progressive decline in cognitive and functional capability, with no cure	Typically, a slower decline in cognitive and functional capability, with no cure
Molecular mechanism	Abnormal protein aggregation, neuroinflammation, oxidative stress, synaptic dysfunction, neuronal loss	Changes in gene expression, epigenetic modifications, oxidative stress, reduced energy metabolism

### 2.1 Overview of APP and Aβ peptides

APP is a transmembrane protein primarily detected in the brain with a large extracellular domain and a short intracellular domain [16]. The β- and γ-secretases cleave APP to produce Aβ. Two pathways are involved in the processing of APP: the non-amyloidogenic and the amyloidogenic pathways. The non-amyloidogenic pathway involves the cleavage of APP by α-secretase within the Aβ domain, to prevent Aβ formation, thereby resulting in the production of a soluble fragment called sAPPα and a membrane-bound fragment called C83. Further cleavage of C83 by γ-secretase generates the P3 peptide and APP intracellular domain (AICD) [17]; whereas, the amyloidogenic pathway leads to the formation of Aβ via APP cleavage by BACE1 at the N-terminus of the Aβ domain to produce a soluble fragment sAPPβ and a membrane-bound fragment C99. C99 then undergoes the cleavage by γ-secretase within the transmembrane domain, leading to the creation of Aβ peptides and the AICD [18]. However, in individuals with AD, these mechanisms are thought to be disrupted, leading to the accumulation of Aβ peptides and the development of Aβ plaques in the brain. Aβ plaques have an essential role in the development of AD, and many studies are ongoing to elucidate the underlying mechanisms of Aβ formation and to develop therapeutic strategies for targeting Aβ [19].

### 2.2 Evidence supporting amyloid cascade hypothesis

The amyloid cascade hypothesis of AD is supported by significant evidence. Histological examination of brain tissues from individuals with AD has shown the presence of Aβ plaques in several areas of the brain. However, these Aβ plaques have not been detected in the brain tissues of healthy individuals [20]. Similarly, many studies have documented a correlation between the presence and density of Aβ plaques in the brain and impaired cognitive function in patients [21]. The existence of Aβ deposition in the brain is a strong predictor of future cognitive decline in patients with slight cognitive impairment, which often precedes the development of AD [21]. Genetic evidence supporting this hypothesis includes the mutations in the gene encoding APP to trigger increased production of Aβ and early onset and familial forms of AD. The mutations in presenilin 1 or 2 and the Aβ sequence of APP change the proteolytic processing of APP and increase the levels of Aβ43 and Aβ42. The studies of underlying mechanisms have demonstrated that the active component of the intramembrane-cleaving c-secretase is encoded by presenilin [22]. The role of presenilin in intramembrane proteolysis is also elucidated in subsequent studies. APP is initially cleaved by endopeptidases near its transmembrane interface and then undergone several carboxypeptidase cleavages to sequentially remove three or four amino acids from the C-terminus. This process yields two product lines that start with either the Aβ49/50 or Aβ48/49 ε-cleavage. Despite differences in specific molecular effects of various presenilin mutations, a similar result of reducing the efficiency of the C- to N-terminal cleavage process can be observed, thereby leading to increased production of longer and more hydrophobic Aβ peptides that can self-aggregate [23].

This sophisticated model offers a biochemical rationale for why harmful mutations in presenilin frequently raise the Aβ42/Aβ40 ratio in humans. Direct γ-secretase reactions performed on AD individual’s tissue with presenilin mutations have documented that mutations could be attributed to reduced carboxy-peptidase-like reactions. Drug-induced activation of carboxypeptidase-like γ-secretase has shown to ameliorate pathogenic effects of mutant presenilin [24]. Additionally, experiments on sporadic AD brains have suggested that non-presenilin-mutant cases may exhibit a similar decline in processivity. Further studies have revealed that Aβ42, Aβ43, and longer Aβ peptides tend to self-aggregate, whereas Aβ40 may possess anti-amyloidogenic properties [25, 26]. Likewise, genetic variation in the apolipoprotein-E (APOE) gene encoding a lipoprotein involved in the clearance of Aβ is strongly associated with the risk of developing late-onset sporadic AD [27, 28]. Overall, these genetic findings strongly support the amyloid cascade hypothesis of AD. Recently, there is increasing interest in exploring the impact of dysfunctional microRNAs within neurons on the occurrence and progression of AD, suggesting that down- or up-regulating microRNAs such as miR-29, miR-124, miR-9 and miR-298 are associated with an increase in Aβ production [29]. However, further investigations are required to be approved for clinical applications.

Several clinical trials of drugs targeting Aβ plaques in the brain, such as solanezumab, have been conducted for the treatment of AD [30]. These drugs are designed to reduce the levels of Aβ plaques in the brain or prevent the formation of Aβ plaques, with the goal of slowing down or reversing cognitive decline in AD patients. In clinical trials, anti-Aβ antibodies are being tested as a class of drugs responsible for binding to Aβ plaques in the brain and promoting Aβ clearance. For example, a clinical study has confirmed that lecanemab as an IgG1 monoclonal antibody has the capability to bind to Aβ soluble protofibrils, thereby reducing Aβ accumulation at the early stage of AD, and resulting in a relatively lower regression in cognition and function than placebo treatment [31]. Another phase III trial of the anti-Aβ antibody gantenerumab has been documented to suppress cognitive decline in patients with prodromal AD [32]. Furthermore, BACE inhibitors, another class of drugs validated in clinical trials, target the β-secretase responsible for suppressing APP cleavage to generate Aβ [33]. Similarly, γ-secretase inhibitors have also been developed and validated in clinical trials for preventing the formation of Aβ [34]. However, substantial debate surrounding the amyloid cascade hypothesis raises questions about the therapeutic efficacy of these drugs targeting Aβ for the treatment of AD.

### 2.3 Limitations of amyloid cascade hypothesis

Although there is significant evidence supporting the amyloid cascade hypothesis of AD, there are several limitations to this hypothesis and considering Aβ as the major cause of AD remains debatable. Similarly, the accumulation of Aβ protein in the brain is a hallmark of AD, however, the molecular mechanisms underlying this process are not fully understood. Clinical trials of drugs targeting Aβ have not successfully alleviated or reversed cognitive decline associated with AD, suggesting that the accumulation of Aβ alone may not be sufficient to cause AD, and other factors may be involved [35]. Furthermore, although Aβ protein is a key component of Aβ plaques in the brain, the accumulation of Tau protein in the brain is also a hallmark of AD. Growing evidence has documented that Tau protein may play a more central role in the development of AD than previous thought [36].

Additionally, it has been observed that up to 40% of elderly individuals without any signs of cognitive impairment can still exhibit some degree of neuropathological features typically associated with AD [37]. Moreover, individuals carrying pathogenic mutations in the presenilin gene are heterozygotes and do not experience any reduction in the function of notch cleavage. Instead, the patients experience an accelerated accumulation of Aβ43 and Aβ42 before the onset of memory impairment. It is worth noting that the majority of patients with AD, including those with familial AD, have normal, unmutated presenilin genes. Therefore, the loss of presenilin function cannot be considered as the major mechanism underlying the pathogenesis of AD [25].

Clinically, one of the major findings is that the degree of cognitive impairment in AD patients does not correlate well with the amount of Aβ plaques in brain tissues, and some patients have significant amounts of Aβ deposits at the time of death, but are not noticeably demented [38, 39]. Considering existing evidence, the amyloid cascade hypothesis cannot be conclusively affirmed, and additional studies are recommended to fully uncover the role of Aβ in AD and to develop more effective treatments for this disease.

## 3. Role of inflammation in AD

Inflammation is increasingly being recognized as a key player in the pathogenesis of AD [40]. The concept of inflammation in AD has been supported by multiple lines of evidence from both *in vivo* models and clinical studies [41, 42]. Previous studies have shown that individuals with AD have elevated levels of inflammatory markers such as cytokines, chemokines, and acute-phase proteins in the brain and cerebrospinal fluid, indicating the presence of chronic low-grade inflammation in the brain [43, 44]. Moreover, the mice that are genetically engineered to develop AD-like pathology have shown increased inflammatory markers and activated microglia in the brain [45-47]. What’s more, AD is more common in individuals with other chronic low-grade inflammatory conditions such as cardiovascular diseases and diabetes [48-50]. The above-mentioned findings support the inflammation hypothesis of AD; however, there are several limitations to this hypothesis. Although the presence of inflammation in the brain tissues of AD patients has been confirmed, it is not clear whether inflammation is a cause or a consequence of this disease, because inflammation is a non-specific response that occurs in many diseases [51]. Moreover, some studies suggest that inflammation occurs early in the disease process and contributes to the development of pathology, while others suggest that it is a secondary response to neuronal damage [52]. Furthermore, some anti-inflammatory drugs have been tested for the management of AD, including but not limited to non-steroidal anti-inflammatory drugs (NSAIDs) and statins, while the results of these trials have been mixed [53]. Overall, although the inflammation hypothesis of AD has generated significant interest, its limitations suggest that it is not the sole explanation for this disease and other pathological mechanisms may also be involved.

### 3.1 Role of microglia and astrocytes in AD

Microglia and astrocytes are two types of glial cells implicated in AD pathogenesis [54, 55]. The risk of AD is linked to genes primarily expressed by glial cells, including microglia, oligodendrocytes, and astrocytes [56]. Microglia possesses a chief role in inflammatory responses, in addition to promoting synaptic plasticity, energy metabolism, and ion homeostasis [57]. Microglia are macrophages of the central nervous system and have been found to exert a chief role in the degradation and internalization of Aβ.

Microglia interact with the fibrillar and soluble forms of Aβ, with the involvement of LDL receptor-related proteins (LRPs)-mediated pathway and macropinocytosis to interact with the soluble forms of Aβ, while the fibrillar type of Aβ activates phagocytosis through initiating intracellular signaling cascades. Inflammatory reactions impact the activation status of microglia and their capability to degrade Aβ [58]. Neurodegenerative processes trigger microglial activation, as observed in the brain tissues of individuals with AD. Compelling evidence regarding microglial roles in AD pathogenesis has been elucidated by molecular biology studies.

Activated microglia have two major types including type M1 and M2 microglia. Type M1 microglia can be induced by lipopolysaccharide (LPS) or interferon-gamma (IFN-γ), and primarily secret pro-inflammatory factors [59]; whereas, type M2 microglia can be stimulated by interleukin (IL)-13/IL-4, and have a phagocytic capacity that is beneficial for supporting neuronal growth and possesses a neuroprotective effect [60, 61]. The long-term triggering of type M1 microglia, in addition to inhibiting type M2 microglia, is thought to be the base for the inflammatory phenotype of AD [62]. The activation of microglia can be triggered by various factors, including peripheral stimulation and the presence of Aβ plaques in the central nervous system in patients with AD [63-65]. Aβ fragments and residues can cause neuronal degeneration and stimulate microglia to produce harmful factors to neurons [66].

In AD, Aβ deposition can activate microglia, thereby culminating in an inflammatory response to stimulate apoptosis. However, this inflammation can also contribute to the further formation of Aβ deposition and turn the inflammation into chronic condition [67].

As oxidative stress is one of the most important hallmarks of AD, microglial reactive oxygen species (ROS) could also cause oxidative stress associated with neurodegeneration. Throughout AD progression, the long-term exposure to damage-associated molecular patterns (DAMPs) causes a noteworthy oxidative stress, endoplasmic reticulum (ER) stress, and the generation of NOD-like receptor protein 3 (NLRP3) in the activated microglia, thereby stimulating the insertion of nuclear factor kappa B (NF-κB) into nucleus, and up-regulating inflammatory genes such as IL-18 and IL-1β to reestablish neural homeostasis [68]. Moreover, APOE has a positive impact on neuronal repair and regeneration by controlling phospholipid and cholesterol distribution and suppressing microglial activity and the corresponding cytokines. This repairing process is similar to the one that occurs after central nervous system injury [69].

**Figure 3.**
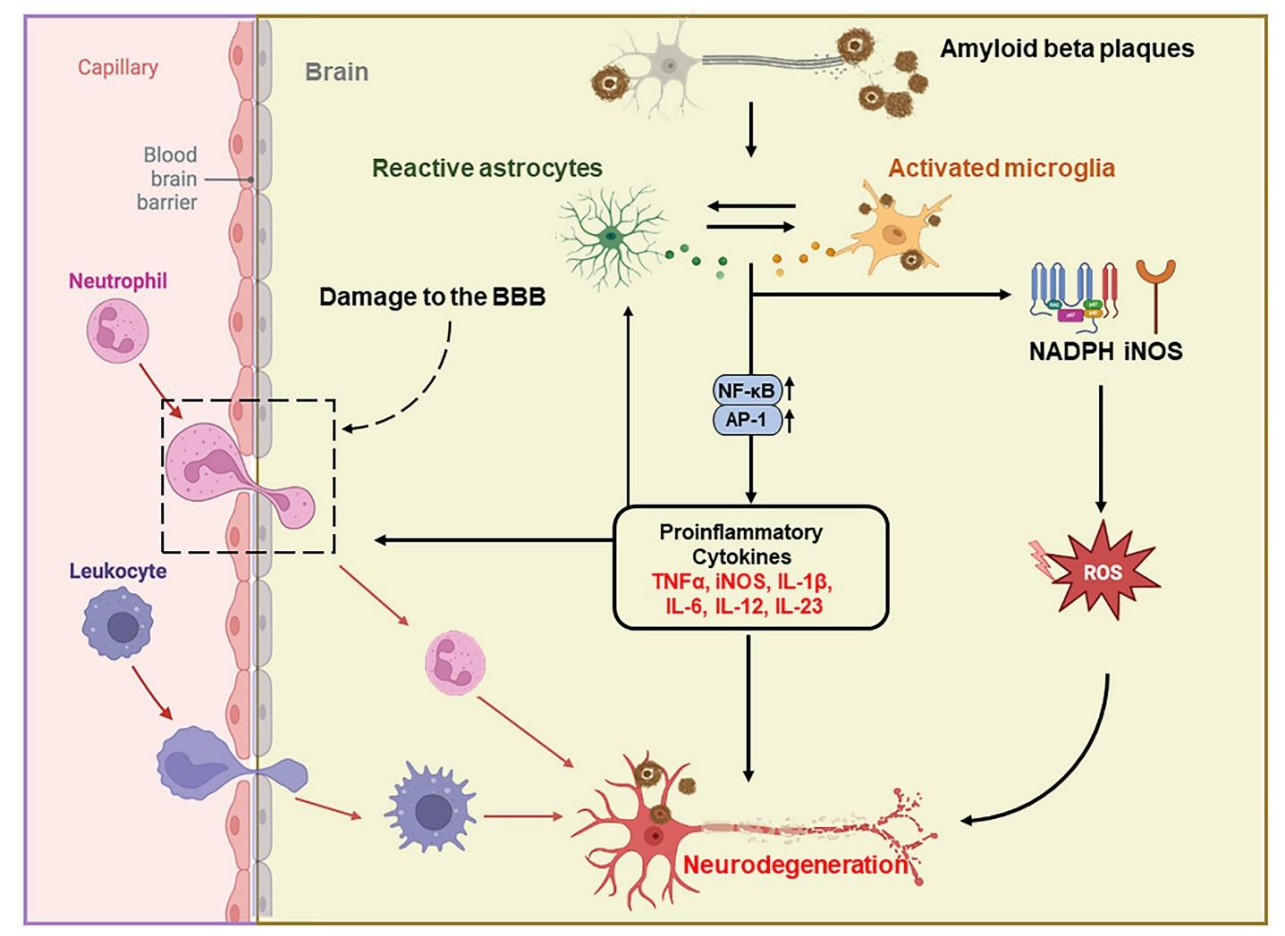
**A diagram illustrating various modes of neuroinflammation in the AD brain for triggering a significant number of Aβ aggregates**. The inability of microglia and astrocytes to phagocytose Aβ aggregates and consequent inflammatory responses lead to the accumulation of Aβ deposits. Aβ aggregates bind to the pattern recognition receptors (PRRs) of microglia to activate downstream target genes, NF-κB, and activated protein 1 (AP-1), thus resulting in the production of cytokines. These cytokines stimulate astrocyte activation and contribute to neuronal damage and neurotoxicity. In addition, the binding of Aβ aggregates to microglia triggers ROS production through NADPH oxidase and inducible nitric oxide synthase pathways, thereby leading to neurotoxicity. Similarly, the binding of Aβ aggregates to astrocyte receptors activates downstream target genes NF-κB and AP-1, thus leading to the production of cytokines. The disrupted communication between neurons, astrocytes, and microglia causes an imbalance in brain homeostasis and promotes neuronal death.

Astrocytes are the most abundant glial cells in the brain, offering metabolic and structural support to neurons, and maintaining the extracellular environment of the brain [70]. It is well known that glial cells, the particular astrocytes, play a critical role in the neuropathology of different neurodegenerative conditions [71]. Astrocytes have shown the capability to clear Aβ, and their dysfunction has been linked to Aβ accumulation in the brain [72]. APOE, a chief genetic risk factor for the late-onset type of AD, is mainly expressed by astrocytes in the brain and stimulates the generation of Aβ [73]. Similarly, other genes primarily expressed by astrocytes are associated with AD, such as clusterin (CLU) and fermitin family homologue 2 (FERMT2). Reactive astrogliosis, as a prominent and early feature of AD, has been detected in animal and clinical studies, and could be earlier to the formation of Aβ plaques [74, 75], suggesting the critical role of astrocytes in the pathogenesis of AD (Fig. 3).

Studies on the brain tissues of patients with AD have shown a close relationship between astrocytes and Aβ deposition [76]. However, this relationship is not fully understood yet. When astrocytes are in the vicinity of senile plaques, they become reactive and display an increase in size and elevated levels of intermediate filament proteins like glial fibrillary acidic protein (GFAP), vimentin, synemin, and nestin [77]. At the molecular level, amyloid-induced astroglial remodeling is activated by calcium release from the endoplasmic reticulum. If this process is inhibited, the astrocytic reactivity is suppressed [78]. However, there is currently no evidence to support astrocytes as the major source of Aβ. Instead, astrocytes appear to play a role in removing Aβ through various mechanisms, such as the expression of AQP4 water channels participating in the glymphatic system. Another mechanism of Aβ clearance is the production of enzymes and proteins involved in the degradation of Aβ, such as neprilysin, insulin-degrading enzyme, matrix metalloproteinases, APOE, and ApoJ/clusterin [79, 80]. Recent studies have also shown that astrocytes isolated from induced pluripotent stem cells (iPSC) of AD patients have a reduced capacity to clear Aβ when compared with healthy controls [81-83]. Additionally, the expression of APOE4 has shown to influence Aβ clearance and seed formation, as well as plaque size, and blood-brain barrier (BBB) integrity, further contributing to cognitive impairment in carriers of the APOE4 allele [84]. Therefore, astrocytes have a crucial role in the nervous system and are important for protecting neurons and maintaining neuronal stability. However, under pathological conditions of AD, astrocytes can undergo different changes that can result in either increased or decreased function, thus leading to neurodegeneration or neuroinflammation. A comprehensive understanding of these cellular changes will enhance our knowledge of how astrocytes change during the occurrence and progression of AD, which could eventually lead to the identification of new biomarkers and therapeutic targets for AD by linking specific astrocyte states to different stages of the disease.

### 3.2 Pro-inflammatory cytokines in AD

Pro-inflammatory cytokines are a group of molecules involved in the inflammatory responses in AD pathogenesis [85]. Many pre-clinical and clinical studies have confirmed the increased production of pro-inflammatory cytokines such as IFN-γ, IL-6, TNF-α and IL-1β, and the activation of relevant receptors in brain tissues of individuals with AD [86]. Moreover, the relationship between increased levels of cytokines and Aβ plaques has also been documented [87].

The molecular mechanism by which pro-inflammatory cytokines activate the NF-κB signaling pathway to trigger AD is also unclear. NF-κB is a transcription factor that controls the expression of a wide range of genes involved in the immune response. In AD, NF-κB is activated by pro-inflammatory cytokines, thus leading to the up-regulation of immune response-related genes and the inflammatory cascade and resulting in the production of additional pro-inflammatory cytokines and the recruitment of additional immune cells to the site of inflammation. Chronic activation of the NF-κB signaling pathway contributes to the progression of AD by promoting neuronal damage and death through further accumulation of Aβ plaques and Tau protein tangles, which in turn perpetuates the cycle of neuroinflammation and neuronal damage.

The generation of pro-inflammatory agents by non-neuronal cells, such as endothelial cells in patients with AD, could be a fundamental factor in AD progression. In the case of AD, brain blood vessels have been found to release more pro-inflammatory agents, including IL-6, IL-1β, and TNF-α, than blood vessels in healthy individuals [88]. The inflammation usually occurs before the appearance of Aβ deposition; however, exposing brain endothelial cells to Aβ promotes the release of additional inflammatory substances [89]. Furthermore, sustained inflammation in peripheral blood circulation could lead to increased pro-inflammatory cytokines within the central nervous system by crossing the BBB. The mechanisms underlying the transport of these cytokines from the bloodstream to the brain have been previously studied [90]. Additionally, Aβ can also cross the BBB to the brain through a process mediated by advanced glycation end products (AGEs) and their receptor (RAGE) [91]. The attachment of Aβ to RAGE on microglia activates microglia, thus leading to increased pro-inflammatory cytokines levels and a sustained inflammatory response [92]. A previous study has shown that Aβ could contribute to BBB dysfunction in patients with cerebral amyloid angiopathy, a condition prevalent among AD patients [93]. This is consistent with a study on triple-transgenic mouse models of AD. Reducing the levels of pro-inflammatory cytokines using a neutralizing antibody can reduce the activity of multiple Tau kinases and the accumulation of Aβ oligomers and phosphorylated Tau [94]. These findings suggest that major inflammatory response within the brain is correlated with high levels of inflammatory cytokines; therefore, these cytokines could be used as biomarkers for local inflammation in AD.

### 3.3 Current studies on inflammation in AD

Current studies on inflammation in AD are mainly focused on understanding the underlying mechanisms of this disease and developing targeted therapies. Researchers are investigating the mechanism by which inflammation contributes to the development of AD and how it changes over the course of the disease. In addition to exploring the relationship between AD and other systemic inflammations, and screening new biomarkers and therapeutic targets for AD, such as specific pro-inflammatory cytokines and signaling pathways involved in the inflammatory response, and developing new anti-inflammatory therapies for AD, such as drugs targeting specific pro-inflammatory cytokines, microglia activation, or other inflammatory pathways [95], some studies are investigating how the immune system can lead to the development of AD [96]. It has become more obvious that the innate immune system has an important role in the progression and pathogenesis of AD [97].

The genes that encode immune receptors such as complement component (3b/4b) receptor 1 (CR1), triggering receptor expressed on myeloid cells 2 (TREM2), cluster of differentiation 33 (CD33), and CLU are associated with the development of AD [98]. Furthermore, progranulin (GRN), pyrin domain containing 1 (PYDC1), B-cell linker protein (BLNK), spleen tyrosine kinase (SYK), solute carrier family 2 member 5 (SLC2A5), and hexosaminidase subunit beta (HEXB) have also been identified as risk genes for the progression and development of AD [99].

Recent studies have shown that the gut-brain axis plays a critical role in AD, and researchers are currently investigating how gut-derived inflammation triggers the development of AD [100]. Differences in gut microbiota, including *Firmicutes, Bacteroidetes, and Bifidobacterium*, between healthy individuals and AD patients, have been detected [101]. Additionally, AD patients with amyloid-positive signs have increased levels of *Shigella* and *Escherichia*, and low levels of *Eubacterium rectale* [102]. These changes could be associated with systemic inflammation, and the gut microbiome perturbation is sometimes associated with elevated proinflammatory microbiota in plasma and bacterial amyloid, which together could lead to systemic inflammation, and hold a potential risk to regulate neurodegenerative conditions [101].

On the other hand, many researchers are investigating the role of environmental factors, such as pollution and diet, in AD and inflammation in the brain [103]. Furthermore, researchers are exploring new targets for anti-inflammatory therapies, such as NLRP3 inflammasomes, as NLRP3 has been linked to the generation of pro-inflammatory cytokines in AD, and anti-inflammatory drugs such as Janus kinase (JAK) inhibitors, which are being tested in clinical trials [68, 104]. Epidemiological evidence has linked to a reduced AD risk during the utilization of anti-inflammatory drugs [3]. Although most therapeutic trials have failed to identify current anti-inflammatory drugs as a cure for AD, substantial evidence from laboratory and epidemiological studies suggests that anti-inflammatory drugs could defer or prevent the occurrence of AD [105].

## 4. Mitochondrial dysfunction and oxidative stress

**Figure 4.**
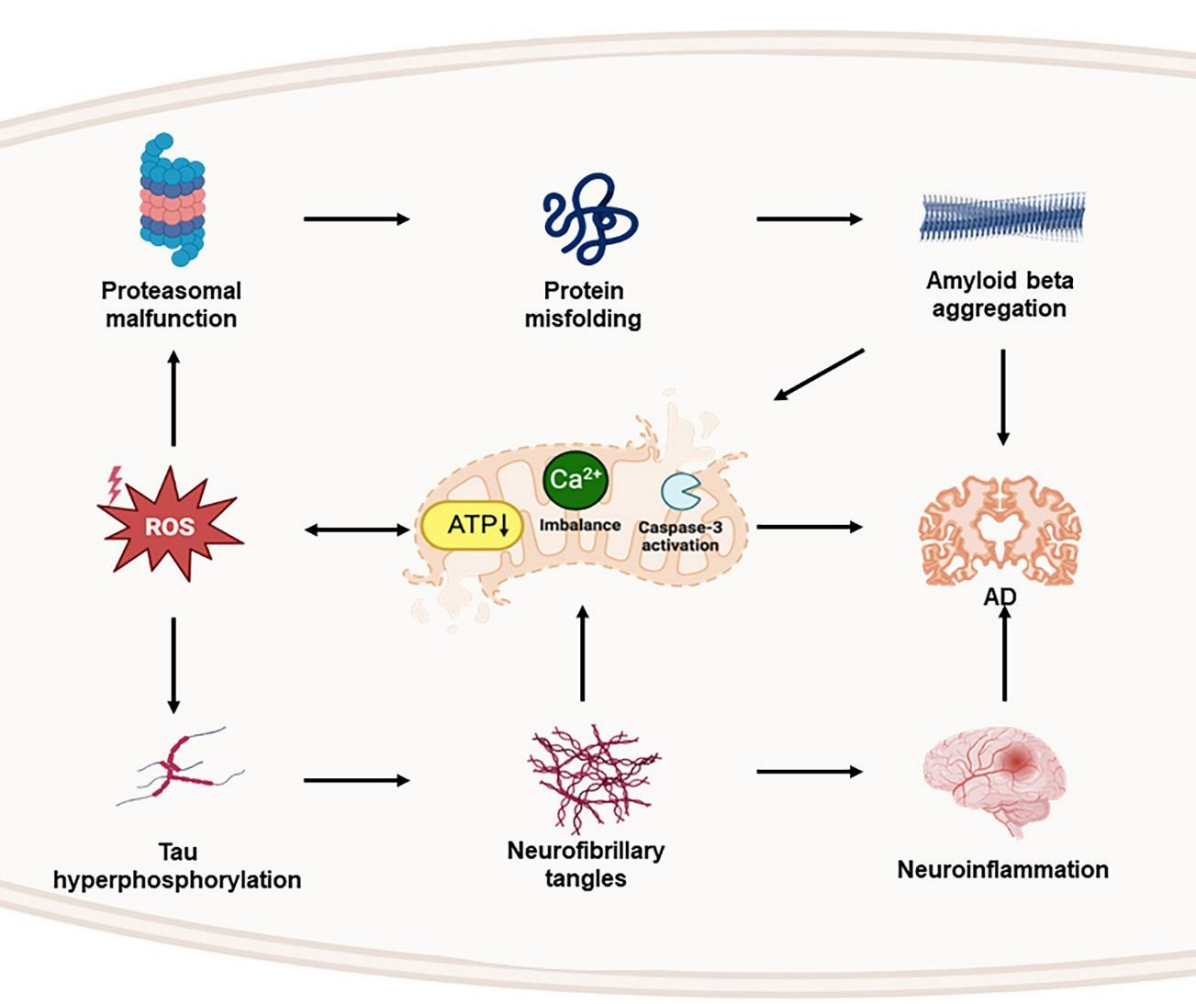
**The complex relationship exists between oxidative stress for triggering mitochondrial dysfunction, and mitochondrial damage for leading to further increased oxidative stress**. Aβ and Tau hyperphosphorylation are also engaged in mitochondrial dysfunction.

Mitochondria are cellular structures that are subjected to constant fission and fusion within eukaryotic cells, and are important for balancing calcium levels, biosynthetic and bioenergetic pathways, and controlling cell death [106]. Mitochondrial dysfunction is a common feature of AD and has been linked to the accumulation of Aβ and NFTs [107]. Aβ affects mitochondrial homeostasis and its enzymatic activity, thereby leading to impaired mitochondrial membrane potential [108, 109]. Therefore, Aβ may lead to severe destructive oxidation due to excessive ROS generation and decreased ATP production [110] (Fig. 4).

It remains unknown whether mitochondrial dysfunction is the cause or a functional outcome of AD. However, the reversal of mitochondrial dysfunction has shown promise as a means of treating AD [111]. Glucagon-like peptide-1 (GLP-1) has shown to have neuroprotective effects in individuals with AD. GLP-1 can ameliorate Aβ-induced energy defects, mitochondrial membrane potential collapse, excessive mitochondrial ROS production, and astrocyte cell toxicity [112]. Moreover, Aβ enters the mitochondria via the translocase of the outer membrane complex, thus leading to exacerbated oxidative stress and inflammation, indicating that mitochondria are also involved in cognitive decline and can be targeted by Aβ [113, 114].

Mitochondrial dysfunction takes place at the early stages of the disease, thus causing an energy deficit for driving the progression of the disease; whereas in the late-onset sporadic AD, mitochondrial dysfunction hypothesis suggests that mitochondrial defect is the primary factor for triggering the accumulation of Aβ and the formation of NFTs, thereby resulting in synaptic degradation and neuronal death [115, 116]. Additionally, neurons require a constant energy supply to function properly and maintain energy homeostasis. Neurons primarily rely on aerobic oxidative phosphorylation to meet energy demands due to limited glycolytic capacity [117]. Mitochondrial oxidative phosphorylation not only offers energy demands of neurons, but also generates ROS, including superoxide radicals (O^2-^) and hydroxyl radicals (OH^-^). The endogenous antioxidant defense system, consisting of catalase (CAT), peroxiredoxin, glutathione peroxidase (GPx), and thioredoxin/thioredoxin reductase, can neutralize ROS [118]. In healthy individuals, a balance exists between the antioxidant defense system and ROS production [119]. However, when excessive ROS are produced, this balance is disrupted, and the antioxidant defense system is unable to neutralize ROS, thereby leading to oxidative stress, and subsequent neuronal damage and mitochondrial dysfunction [120, 121]. The generation of excessive ROS can cause significant destruction to essential mitochondrial components such as proteins, DNA, and lipids [122]. Moreover, mitochondria are particularly vulnerable to oxidation due to the absence of histones [119]. The vulnerability of oxidation process results in a limited capacity and fidelity of mitochondrial DNA repair.

Brain positron-emission tomography (PET) imaging can reveal the changes in energy metabolism, including decreased glucose consumption by neurons and diminished enzymatic activity of mitochondrial components [123]. When ATP production in mitochondria decreases, extracellular signal-regulated kinases 1 and 2 (ERK1/2) are activated to stimulate the hyperphosphorylation of Tau protein and the formation of paired helical filaments, thereby contributing to the development of AD [124].

Furthermore, mitochondrial dysfunction has been proposed as a therapeutic target in AD. Several treatments focus on targeting mitochondrial function, such as antioxidants and anti-inflammatory agents. The pre-clinical studies have shown that AD progression in mouse and rat models can be slowed down by reversing mitochondrial dysfunction through the targeted delivery of antioxidants [125]. However, the relationship among AD, oxidative stress, and mitochondrial dysfunction is complex, and is not fully understood yet.

On the other hand, there are several limitations to this hypothesis. First, whether mitochondrial dysfunction and oxidative stress are the cause, or the outcome of the disease is still debated. Mitochondrial dysfunction and oxidative stress are also observed in other neurodegenerative diseases, as well as in normal aging. Therefore, it is unclear whether these factors are specific to AD or represent general features of neurodegeneration [126]. Moreover, some studies have demonstrated that antioxidant supplementation, aimed at mitigating oxidative stress, does not significantly alter the progression of AD, which raises questions about therapeutic implications based on this hypothesis [127].

### 4.1 Role of energy metabolism in AD

Energy metabolism is the process for converting nutrients into energy. Growing evidence over the last few years suggests that energy metabolism disorders in the brain are the early and chief pathogenesis of AD. Several risk factors for energy metabolism disorders have been identified, such as Aβ plaques, mitochondrial dysfunction, insulin resistance, oxidative stress, neuroinflammation, aging, and NFTs [128]. Recently, numerous studies have determined a relationship between metabolic disorders and AD [129, 130]. The altered energy metabolism has been associated with aging and the pathogenesis of late-onset AD [131]. Diabetes and obesity show a role in increasing the risk of AD and cognitive decline, suggesting the impairment in brain glucose metabolism [132].

Additionally, AD is associated with disruptions in central and peripheral insulin signaling, including changes in insulin-like growth factor (IGF) and brain insulin levels [132]. The changes in insulin signaling impact memory functions, neuronal survival, gene expression, and energy balance [133-135]. Phosphorylation of Tau protein can be triggered by kinase activation in the presence of insulin and IGF1 [136-138]. As a result, AD may be considered as "type 3 diabetes", as insulin serves as a connection between these two chronic diseases. Earlier studies have found that insulin plays a regulatory role in neuronal function in the cortex and hippocampal tissues [136-138]. Furthermore, soluble Aβ oligomers have been shown to modify insulin signaling by binding to insulin receptors in hippocampal neurons, resulting in the migration of receptors from the membrane into the cell [130].

Earlier research has highlighted the association between Aβ oligomers/fibrils and the development of neuronal insulin resistance in AD. To mitigate this effect, intranasal insulin therapy is currently being investigated as a potential therapeutic approach for AD. However, an opposite relationship has also been observed, as elevated insulin levels have been detected in AD patients. Furthermore, studies have demonstrated that high insulin levels can promote Aβ accumulation and Tau phosphorylation, potentially aggravating AD pathology [139].

An increasing body of studies has connected changes in mammalian target of rapamycin (mTOR) and serine/threonine kinases to aging-induced cognitive decline and the development of AD [140]. The mTOR possesses a crucial role in memory maintenance, synaptic adaptability, and neuronal repair in the nervous system. However, disruptions in mTOR have been linked to numerous diseases, including cancer, metabolic diseases, and neurological diseases [140], suggesting that abnormal mTOR signaling in the brain can affect numerous pathways related to mitochondrial function and energy metabolism [141].

### 4.2 ROS accumulation in AD

ROS are a group of molecules produced as a byproduct of regular cellular metabolism and involved in the pathogenesis of AD. Oxidative stress occurs due to the imbalance between ROS level and neutralization capability in cells. Numerous studies have demonstrated that increased oxidative stress has an important role in triggering pathological changes during the early and symptom-free stages of AD [142, 143]. The structure, fast metabolism, and high lipid levels in the brain make it vulnerable to the damaging consequences of oxidative stress [144]. Oxidative stress in the cortex and hippocampal tissues is a common characteristic of AD [145]. An experiment on mice lacking Cu/Zn superoxide dismutase (SOD) has shown that the high rate of oxidative stress, especially in the hippocampal tissue, causes a decline in cognitive function closely resembling the decline observed in the model of aged mice. Moreover, low levels of antioxidant enzymes are detected in animal models and individuals with AD [146]. Therefore, controlling oxidative stress through using antioxidants may be a potential strategy for treating this disease. Increased turnover of ROS resulting from impaired regulation can ultimately give rise to oxidative stress in specific brain regions that are prone to AD neuropathology, thereby leading to dysfunction in neuronal mitochondria and synapses [147].

A protein carbonyl alteration in cerebrospinal fluid has also been observed during the mild cognitive impairment stage [148]. The cases with mild cognitive impairment have revealed considerably higher levels of oxidative alterations in biological macromolecules of the hippocampal tissue [149]. Meanwhile, oxidative stress is known to cause modifications of the lipid bilayer and the increased levels of 4-hydroxy-2-nonenal in both cerebrospinal fluid and brain tissue of individuals with AD [150]. The increased protein carbonyl content in specific regions that are abundant in senile plaques suggests a possible link between oxidative damage and the development of AD lesions [151].

The suppressed glucose metabolism in the brain, as revealed by fluorodeoxyglucose-positive emission tomography analysis, implicates the involvement of mitochondria in the pathogenesis of mild cognitive impairment and AD [152]. Intriguingly, the reduction in glucose metabolism is poorly correlated with the presence of Aβ plaques or NFTs [153].

Mounting evidence suggests that the damage to synapses represents an early occurrence during the development of AD, and synaptic impairment corresponds to memory weakening in early AD, even before neuronal damage [154]. The spread of synaptic loss and phosphorylated Tau pathology in other brain regions could occur via a synaptic mechanism [155]. Therefore, it is plausible that dysfunctional synapses in AD may be due to oxidative stress and insufficient antioxidant quenching mechanisms [156]. Moreover, synaptic deficits are associated with N-methyl-D-aspartate (NMDA) and α-amino-3-hydroxy-5-methyl-4-isoxazole propionic acid (AMPA) receptor-mediated excitotoxicity, as well as inadequate compensatory GABAergic neuronal circuitry [157, 158]. During the early stages of AD and mild cognitive impairment, Aβ plaques stimulate the degeneration of synaptic membranes, thus causing long-term cognitive impairment and subsequently affecting memory and learning capacity [159]. As a result, oxidative modifications, defective glucose metabolism, and insufficient ATP synthesis by mitochondria may result in imbalanced calcium signaling, abnormal membrane potential, impaired neurotransmission, and abnormal action potential firing. The functional and cellular changes cause structural changes in dendrites and synapses, thus resulting in reduced executive function, memory and learning defects, and weakened reasoning in patients with mild cognitive impairment and AD [147].

Pre-clinical studies have clearly revealed a surge in oxidative stress in the cortex and hippocampal tissues of the brain with the progression of AD. Local infusion of oxidizing agents into the hippocampal tissue of model mice can increase local Aβ42 levels in the interstitial fluid [160], whereas the administration of the Aβ42 peptide into the forebrain of model rats can produce oxidative stress in the hippocampal tissue [161]. These experimental findings suggest that oxidative stress plays an upstream role in AD. Moreover, the levels of lipid peroxidation markers, including 8,12-iso-iPF2a-VI, in plasma, brain tissue, and urine samples, are significantly high in both transgenic AD model mice and AD model mice induced through intracerebroventricular streptozotocin injection [162, 163]. Furthermore, the surged level of oxidative stress is associated with reduced levels of antioxidant enzymes such as CAT and SOD [164]. In summary, oxidative stress possesses a crucial impact on AD. However, using antioxidants to treat AD still requires further investigations.

### 4.3 Mitochondrial defects and oxidative stress in AD

The mitochondrion contains a porous external mitochondrial membrane that enables uncharged molecules and tiny ions to move freely, as well as an impermeable inner membrane that surrounds the mitochondrial matrix. The inter-membranous gap exists between the two membranes [165]. The mitochondrial electron transport chain is made up of multiple complex proteins that are located in the inner mitochondrial membrane. These complexes use electrons released by reduced flavin adenine and nicotinamide adenine dinucleotides from Krebs cycle to pump protons from the matrix into the intermembrane gap, which generates a proton gradient across the inner mitochondrial membrane, thus facilitating the production of ATP [166].

The folded inner mitochondrial membrane must assemble these complexes into specially designed structures to function appropriately [167]. However, roughly 1-2% of the utilized oxygen, even under ideal circumstances, leaks and produces ROS [168]. There are at least eight locations in the mitochondria that produce ROS [165, 169]. The produced ROS can affect mitochondrial function and cause neuronal degeneration through DNA damage, protein oxidation, and lipid peroxidation [170, 171].

Regarding this event, mitochondrial dysfunction induced by abnormal ROS processing is a critical component in the etiology of AD [172]. Similarly, the introduction of Aβ oligomers into the bilayer may result in the formation of ROS, which can initiate membrane lipid peroxidation and subsequently cause oxidation of intracellular proteins and nucleic acids [173, 174]. Therefore, reducing ROS levels by various interventions, including exercise, antioxidant medications, and diet, could shield mitochondria from oxidative damage and hence reduce the risk of AD [175].

## 5. Tau hyperphosphorylation hypothesis

**Figure 5.**
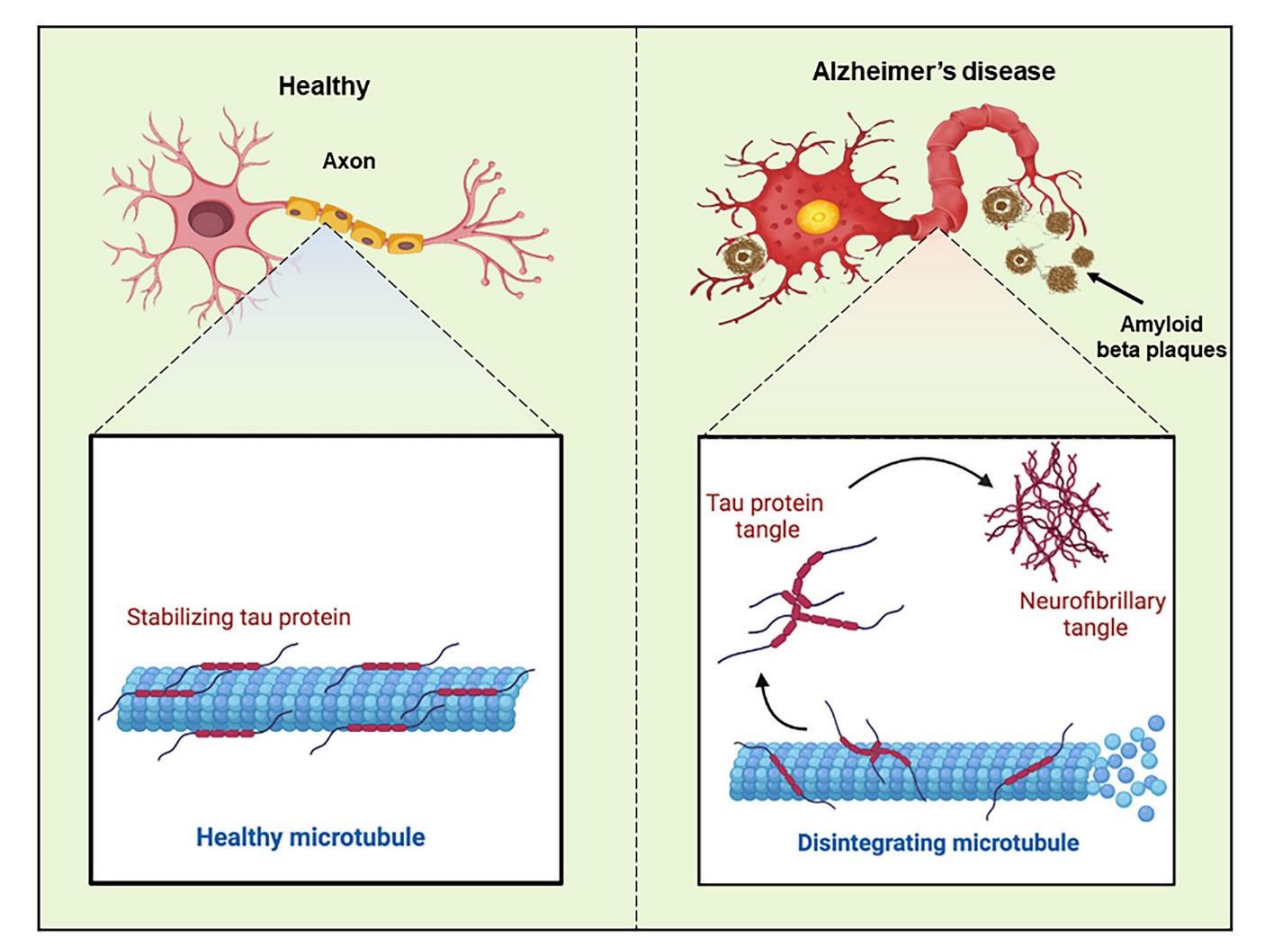
Schematic diagram describing the healthy and AD neurons according to Tau hypothesis.

Tau protein, a phosphoprotein, binds to and stabilizes microtubules. The microtubules provide a framework for the transport of molecules within neurons [176]. Tau protein is essential for preserving the structure and functionality of neurons. Tau hypothesis proposes that the accumulation of abnormal Tau protein in the brain is the major driver of the disease [177] (Fig. 5).

The aggregation of Tau in entorhinal cortex and brainstem is the earliest observed event in AD. With the progression of AD, Tau spreads to other regions of brain [178]. The animal model studies have shown that abnormal Tau protein is transferred from neuron to neuron through a trans-synaptic mechanism, thus resulting in accumulation of NFTs and subsequent neurodegeneration [179]. The updates on the Tau hypothesis take a more nuanced view of the role of Tau protein in the disease. While NFTs are the hallmark of the disease, the updated hypothesis recognizes that there are multiple forms of Tau protein, and not all forms are equally harmful. For example, recent research has suggested that small, soluble forms of Tau may be more toxic than larger, aggregated forms [180]. The updated hypothesis also acknowledges that Tau pathology does not occur in isolation, and it may interact with other factors, such as inflammation and genetics factors [181].

Although both Aβ plaques and Tau tangles have been identified as crucial factors in AD, recent research suggests that the biological pathways of AD can lead to Tau pathology independent of Aβ. This highlights the potential importance of the Tau hypothesis as a complementary approach to the Aβ hypothesis. These results imply that Aβ-reducing therapy may not be enough to stop Tau pathology and Tau-mediated neuro-degeneration from progressing in patients [182]. On the other hand, emerging evidence has shown that the correlation between cognitive impairment and phosphorylated Tau aggregates is stronger than its correlation with Aβ aggregates [183], and the number and distribution of NFTs are closely correlated with the severity of cognitive function decline [184]. In cases where NFTs are detected in the absence of Aβ aggregates, NFTs are described as primary aging-related Tauopathy, which mostly occurs in the early, pre-amyloid phase of the AD pathogenesis [185].

### 5.1 Overview of Tau protein and NFTs

Tau protein has six isoforms that are generated by alternative splicing of the Tau gene, and involves in stabilizing the structure of neurons and regulating their internal transport systems [186]. NFTs, the abnormal clumps of Tau protein, are considered one of the hallmark features of AD and are composed of Tau protein aggregates accumulated in the brain of patients. Accumulated NFTs can disrupt normal function of neurons, ultimately resulting in cognitive impairment and other symptoms associated with AD.

The formation of pathogenic Tau can be triggered by a variety of factors, including brain injuries, TOR pathway hyperactivity, mutations in the Tau gene, and metabolic syndrome [187]. Moreover, the accumulation of Aβ within neurons can activate kinases, such as glycogen synthase kinase 3-beta (GSK-3β), which can phosphorylate Tau protein and lead to its detachment from microtubules, destabilization of the cytoskeleton, and subsequent formation of NFTs, thereby leading to apoptosis or cell death [188].

### 5.2 Evidence supporting Tau hyperphosphorylation hypothesis

Growing evidence supports the Tau hyper-phosphorylation hypothesis of AD. A noteworthy number of studies have convincingly demonstrated the presence of NFTs in brain tissues of AD patients including neocortex, hippocampus, and entorhinal cortex. NFTs consist of hyperphosphorylated Tau, and the phosphorylation of Tau can decrease its capability to bind to microtubules, thus leading to the accumulation of NFTs [189].

Earlier studies have provided evidence that NFTs are closely associated with cognitive decline of AD patients [190]. Moreover, genetic studies have shown that mutations in the Tau gene are associated with AD [191]. Mutations in the microtubule-associated protein Tau (MAPT) gene that increases the aggregation of Tau protein have been linked to frontotemporal dementia and parkinsonism in the chromosome 17 (FTDP-17). Therefore, Tau dysfunction is sufficient to cause neurodegeneration and dementia [192].

Moreover, some drugs are designed to target Tau protein, such as Tau-directed monoclonal antibody (ABBV-8E12), which can reduce the level of Tau protein in cerebrospinal fluid, but no significant improvement in cognitive function is achieved when compared with the placebo group [193]. Finally, the level of Tau protein in cerebrospinal fluid and blood can be used as a biomarker to diagnose AD and monitor the progression of the AD [194].

### 5.3 Limitations of Tau hyperphosphorylation hypothesis

Although the evidence supporting the Tau hyperphosphorylation of AD is relatively strong, there are some limitations to this hypothesis. First of all, not all cases of AD show high levels of NFTs, suggesting that Tau pathology alone may not be sufficient to cause AD and other factors may also be involved in the development of AD [195]. It is known that Tau protein becomes abnormal and forms NFTs in AD; however, the underlying mechanisms are not fully understood.

Additionally, abnormal Tau protein has also been found in other neurodegenerative diseases like progressive supranuclear palsy and corticobasal degeneration, suggesting that Tau pathology is not specific to AD and the underlying mechanisms might be different [196]. It is necessary to know that despite the overwhelming evidence for the Tau hypothesis of AD, it is not the only explanation for the underlying mechanism. Recent studies suggest that both Aβ plaques and Tau tangles are important drivers of AD, and may interact with each other in a reciprocal manner [197]. Despite the abovementioned limitations, Tau hypothesis remains a key concept in AD, and further studies are needed to fully elucidate the pathogenesis of AD.

## 6. Calcium signaling hypothesis in AD

Calcium is essential for controlling a number of processes, including synaptic plasticity, neurotransmitter transmission, and learning and memory capacity. Unbalanced cellular calcium could lead to pathophysiological conditions such as apoptosis, degeneration, autophagy deficit, and necrosis [198]. Several studies on AD models suggest a link between the neurobiology of AD and disturbances in intracellular calcium homeostasis [199]. The calcium signaling hypothesis of AD proposes that dysregulated calcium signaling is a critical factor in the development of AD by exacerbating the hyperphosphorylation of Tau protein and deposition of Aβ, in addition to anomalous synaptic plasticity, all of which are fundamental hallmarks of AD. Neuronal calcium homeostasis is firmly controlled through a complex interplay between pumps, exchangers, channels, and buffering proteins [200]. The key players in neuronal calcium regulation include: NMDA receptors, voltage-gated calcium channels (VGCCs), ryanodine receptors (RyR), inositol 1,4,5-trisphosphate receptors (IP3Rs) in the endoplasmic reticulum, plasma membrane calcium ATPase (PMCA), sodium-calcium exchangers (NCXs), and the mitochondria as calcium buffers [201].

The Ca^2+^ dysregulation has shown to be an early event of AD. The calcium signaling hypothesis suggests that activation of the amyloidogenic pathway disturbs the neuronal Ca^2+^ signaling pathway, which in turn, disturbs Ca^2+^ homeostasis and results in impairment of learning and memory capacity. Furthermore, the Ca^2+^ signaling hypothesis of AD proposes that hyperphosphorylated Tau affects Ca^2+^ homeostasis because it disrupts neuronal circuits, activates VGCCs, and reduces nuclear Ca^2+^ [199].

The concentration of calcium ions in neurons located near Aβ deposits is higher than the normal resting level [202]. Elevated calcium concentration can promote negative plasticity [203], which is triggered by an increase in the expression and activity of calcineurin (CaN), a protein activated by calmodulin (CaM), in response to the small increase in intracellular calcium levels. When CaN is activated, it triggers the activation of additional phosphatases, such as protein phosphatase 1 (PP1), which further induces long-term depolarization (LTD) that can erase memory [204]. Conversely, when Ca^2+^/CaM binding is disrupted, the Ca^2+^/calmodulin-dependent protein kinase II (CaMKII), which plays a crucial role in synaptic plasticity and memory formation, can be activated [205]. CaMKII is responsible for Tau phosphorylation, while calcineurin is involved in Tau dephosphorylation. The dysregulation of these enzymes due to calcium imbalance can lead to abnormal Tau phosphorylation and aggregation [206]. Furthermore, Aβ oligomers can elevate the cytosolic calcium concentration through various mechanisms, such as by forming novel pores on plasma membrane or by stimulating metabotropic glutamate receptor 5 (mGluR5), which increases the creation of inositol 1,4,5-trisphosphate (InsP3) and subsequently leads to calcium release [207, 208]. The intracellular Aβ oligomers can also stimulate G-protein-mediated calcium release from the endoplasmic reticulum (ER) via InsP3 [209]. The ER is an organelle that actively clears calcium ions from the cytoplasm and discharges stored calcium ions into the cytosolic compartment via calcium channel receptors, such as InsP3R or RYR. Enormous calcium release from the ER via activation of InsP3R and/or RYR is linked to Tau and amyloid deposition, which contributes to memory and learning deficits in AD [210]. RYR can be activated by calcium ions themselves and may enhance the function of InsP3R by calcium release mechanism [211]. The reduction in ER calcium ions may result from this process, thus leading to a decrease in the production of vacuolar-type H^+^-ATPase (vATPase). The reduction in vATPase production is attributed to a protein-folding reaction that requires a high concentration of calcium ions within the ER [212]. Disruption in the maturation of vATPase within the ER can lead to a decrease in vATPase production, which impairs the maintenance of proper pH in lysosomes. This condition results in compromised lysosomal function and acidification, ultimately leading to autolysosome and dysfunctional autophagy. The implications of such events can be observed in the development of AD. Notably, abnormalities in ER calcium signaling occur before the detection of Aβ and Tau in AD cases [213].

The implications of AD-caused abnormal homeostasis of calcium ions spread beyond the ER. Mitochondria are closely attached to the ER via the mitochondria-associated ER membrane. Disturbances in Ca^2+^ homeostasis, particularly mitochondrial Ca^2+^ homeostasis, can have detrimental effects on function and survival of cells. In AD, there is a reduction in ATP level, an increase in ROS production, and impaired mitochondrial function, all of which are linked to the alterations in mitochondrial Ca^2+^ homeostasis [214]. Notably, peripheral tissues from AD patients also exhibit mitochondrial damage, as demonstrated by defective mitochondrial dynamics and bioenergetics, as well as abnormal Ca^2+^ homeostasis in fibroblasts [215]. Furthermore, abnormal mitochondrial Ca^2+^ homeostasis in AD can lead to other mitochondrial Ca^2+^-related disturbances, such as alterations in mitochondrial dynamics, mitochondrial Ca^2+^ buffering, and mitophagy [215]. These processes have been proposed to be involved in the pathogenesis of AD, and their dysregulation can contribute to disease progression. Moreover, APP and Aβ are known to accumulate in the mitochondrial matrix. The presence of Aβ affects mitochondrial Ca^2+^ levels. When neurons are directly exposed to soluble Aβ oligomers *in vitro*, mitochondrial Ca^2+^ uptake occurs, thereby leading to mitochondrial Ca^2+^ overload. The overload of mitochondrial Ca^2+^ activates the mitochondrial permeability transition pore (mPTP), which then causes cytochrome C release and apoptosis [216].

The reduced expression of the Na^+^/Ca^2+^/Li^+^ exchanger (NCLX), which is responsible for the Ca^2+^ efflux from mitochondria, is another proposed mechanism for Aβ-mediated mitochondrial Ca^2+^ overload. Recent studies have reported to have decreased NCLX expression in human AD brains and AD mouse models, which could contribute to the progression of the disease by causing impaired mitochondrial Ca^2+^ efflux and overload [217].

Presenilins have been suggested to play a role in regulating mitochondrial Ca^2+^ homeostasis and synaptic plasticity. Studies have proposed that presenilins act as low-conductance endoplasmic reticulum leak channels, thus contributing to maintaining physiological Ca^2+^ levels within the ER [218]. However, familial AD-linked mutations in presenilins could result in a loss of function of the ER Ca^2+^ leak channel and impair Ca^2+^ release from the ER, thus leading to the accumulation of Ca^2+^ in this organelle and an increased predisposition to degeneration [219]. Nevertheless, other studies have failed to support these observations and have suggested that familial AD presenilin-linked mutations reduce, rather than increase, ER and Golgi Ca^2+^ levels [220].

As a result of abnormal hyperphosphorylation and misfolding, Tau detaches from microtubules, accumulates, and reaches the somatodendritic compartment, interrupts mitochondrial transport, deprives energy, causes oxidative stress, and finally leads to neurodegeneration [221]. Increased cytosolic calcium leads to Tau hyperphosphorylation by triggering microsomal prostaglandin E synthase 1 (mPGES1) or kinases [222]. Aβ oligomers have been demonstrated to raise intracellular calcium levels through multiple mechanisms, such as the formation of calcium-permeable pores in cell membranes, activation of NMDA receptors, and modulation of VGCCs. Furthermore, Aβ can trigger ER stress, which can result in increased calcium release through RyRs and IP3Rs [199, 223]. Aβ can also increase the postsynaptic calcium levels by activating mGluR5 receptors. The mGluR and NMDA receptors promote APP processing, thus leading to increased ROS generation and calcium influx in a positive feedback loop [224].

Numerous therapeutic drugs that target plasma Ca^2+^ channels have shown promising efficacy in pre-clinical studies. Among these drugs, memantine has already gained FDA approval for the treatment of AD. However, it is important to note that the majority of developed drugs primarily target the plasma membrane channels, rather than the intracellular calcium signaling pathways that are most commonly disrupted in AD, such as ER and lysosomes [225].

On the other hand, there are several limitations to this hypothesis. Calcium signaling is a complex process, and it is challenging to identify precise molecular mechanisms underlying its role in AD. Furthermore, AD is a multifaceted disease with multiple contributing factors, and the calcium signaling hypothesis may not account for all of these factors. While preclinical studies have shown promise for calcium modulators as potential AD treatments, clinical trials in humans have yielded mixed results. Some studies have reported modest benefits, while others have failed to replicate them [226]. Moreover, calcium modulators can have side effects due to the ubiquitous role of calcium in cellular processes, making their use as an AD treatment challenging. Furthermore, it is unknown whether abnormal mitochondrial Ca^2+^ homeostasis leads to AD or it is a result of Aβ and pathological Tau aggregation, and the precise mechanisms are not yet fully understood. Therefore, further research is necessary to fully comprehend the role of impaired calcium regulation in AD and develop safe and effective therapeutic approaches.

## 7. Cholinergic hypothesis

The cholinergic hypothesis, one of the hypotheses proposed to explain the development of AD, suggests a central role for the dysfunction of cholinergic neurotransmission in the pathogenesis of AD. This hypothesis is based on the observation that there is a significant loss of cholinergic neurons and a reduction in acetylcholine (ACh) levels in the brains of patients with AD [227]. ACh is involved in various cognitive functions such as learning and memory capacity, and higher-level behaviour [228]. In particular, basal forebrain cholinergic neurons (BFCNs) have a major role in memory, learning, and cognitive functions. The differentiation and survival of BFCNs are mainly linked to the nerve growth factor (NGF), to exert their influence over key brain regions involved in memory and learning capacity, such as the cortex and hippocampus [229]. AD patients experience degeneration of BFCNs, which correlates with the severity of dementia. This degeneration process contributes to synaptic loss between the basal forebrain and corresponding tissues, including the hippocampus and cortex, thereby leading to memory impairment, a hallmark symptom of AD [230].

Clinical observations have revealed a reduction in the number of cholinergic neurons due to significant neurodegeneration, and obvious deficiency in ACh transferase (AChE) activity in the brains of AD patients. These findings highlight the substantial damage inflicted upon the cholinergic system in AD [231]. Consequently, therapeutic approaches aiming to restore the cholinergic system have gained prominence in clinical practice. Another aspect worth considering is the interaction between AChE and PS-1, a key enzyme involved in Aβ production. AChE binds directly to PS-1 for enhancing its expression and subsequently increasing the level of Aβ [232]. The accumulation of Aβ is known to accelerate cognitive dysfunction in AD [233]. Additionally, abnormal changes in the central cholinergic system can stimulate inflammation in nerve cells and the phosphorylation of Tau protein, thereby resulting in the imbalanced neurotransmitters and neuronal apoptosis. However, the precise mechanisms by which these processes occur in relation to AD are still not fully understood. It is important to recognize that AD not only inflicts irreversible and severe damage to the quality of life and mental well-being of patients, but also imposes significant economic and medical burdens on their families and society as a whole [234].

The cholinergic hypothesis has been supported by several lines of evidence, including the correlation between cognitive decline in AD patients and the reduction in cholinergic markers, such as choline acetyltransferase (ChAT) activity responsible for ACh synthesis [235]. Moreover, cholinesterase inhibitors (ChEIs), such as tacrine, and metrifonate, have been shown to improve cognitive function in AD patients. ChEIs increase ACh levels by inhibiting its degradation [236]. The cognitive impairments can be observed in animal models of AD after pharmacological or genetic manipulation of the cholinergic system [237, 238].

The cholinergic hypothesis is not without its limitations. Although ChEIs provide symptomatic relief, they do not halt or reverse the progression of the disease, and they only have a modest effect on AD progression [238]. In addition, other neurotransmitter systems are also affected in AD, and there is growing evidence that pathological changes in AD, such as Aβ plaques and Tau neurofibrillary tangles, can lead to synaptic dysfunction and neuronal loss. Therefore, the cholinergic hypothesis has been an influential theory in AD studies, thereby leading to the development of ChEIs for AD treatment.

## 8. Vascular hypothesis of AD

The vascular hypothesis is born out of epidemiological studies that have demonstrated a significant association between cardiovascular disease and the onset and progression of AD [239]. Vascular risk factors such as diabetes, hypertension, and hyperhomocysteinemia are strongly linked to an increased likelihood of AD. These risk factors are known to cause disruption of the BBB, hemodynamic changes, and a reduction in cerebral blood flow, thus leading to cerebral hypoperfusion-hypoxia, which further contributes to the development of AD [240]. However, the role of vascular risk factors in AD development remains unclear. Pathological investigations have shown smaller capillaries, fewer cerebral microvessels, and denser capillaries in AD brains when compared with normal ones [241]. In addition to the atrophy of smooth muscle cells in cerebral vessels and the impairment of capillary endothelium, the rupture and bleeding of intracerebral vessels have also been observed [242]. Although Aβ is observed in blood vessels of meningeal and gray matter, it is also detected in the cortical and subcortical gray matter [243]. A previous neuropathological study has shown that 92% of individuals with AD have cerebral arteriosclerotic alterations [244]. Moreover, the Willis circle and the major leptomeningeal arteries have been found to have atherosclerosis [245].

Cerebral hypoperfusion is a preclinical condition and has been identified as one of the most reliable predictors of individuals progressing to AD. Perfusion single-photon emission computed tomography (SPECT) has demonstrated that patients with cognitive deficits in AD typically have reduced regional perfusion in the posterior cingulate, posterior parietal, and temporal lobe regions, whereas AD patients with psychologic and behavioural symptoms have different perfusion patterns [246]. The pathogenesis of AD may be significantly influenced by the decreased cerebral blood flow [247]. The cholinergic neuron loss and Aβ production may be the results of a dysfunctional cerebrovasculature, which initiates from chronically inadequate blood flow to the brain [248]. For example, decreased cerebral blood flow in aged rats can result in neurodegeneration and deficits in metabolism and memory that resemble the pathophysiology observed in AD [249]. Additionally, during ischemia, the mitochondria of vulnerable cells invariably exhibit damage [247]. Moreover, the chronic hypoperfusion may stimulate mitochondrial malfunction in vascular cells, thereby triggering the generation of ROS [250]. The amount of ROS is greater in the aged and damaged mitochondria. In the case of respiratory inhibition due to the reduction in the regional cerebral blood flow, the O_2_ transforms into superoxide O_2_ [251]. ROS aggregation has the capability to destroy biomolecules including nucleic acids, proteins, and lipids. The brain is more likely to experience lipid peroxidation than other organs due to the higher amount of polyunsaturated fatty acids and lower activity of antioxidant enzymes such as catalase and glutathione peroxidase [252]. Nitric oxide (NO) and superoxide may combine to generate peroxynitrite, which is extremely harmful to DNA and RNA. The reduction of NO, a vasodilator, is caused by elevated ROS levels. Therefore, elevated oxidative stress prevents NO from functioning and vasodilation. This may play a key role in the vascular anomalies underlying metabolic dysregulation in AD [247]. Furthermore, oxidative alterations to mitochondrial DNA have the potential to result in bio-energetic impairments, thereby eventually leading to neuronal death [253]. When cellular antioxidant defences are unable to maintain ROS levels below a hazardous threshold [252], increased levels of oxidative stress, including RNA, DNA and protein oxidation, and lipid peroxidation, are present in AD brains [252]. According to a previous report, vascular lesions and mitochondrial characteristics observed in vascular wall cells from human AD brain biopsies serve as the indicators of oxidative damage. Furthermore, there is a higher deposition of Aβ in the vascular walls of AD patients when compared with age-matched individuals. Additionally, in the vascular endothelium and perivascular cells of microvessels with atherosclerotic lesions, a higher amount of 8-hydroxyguanosine (8-OHG) and noticeably more abnormal mitochondria are observed, but these characteristics are not observed in AD brain tissues that are not injured or in age-matched normal individuals [253], suggesting that, prior to the appearance of AD pathology, vascular wall cells could be the major target for oxidative damage [247].

Mesenchyme homeobox 2 (MEOX2), a homeodomain transcriptional factor, contributes to smooth muscle cell migration and angiogenesis. MEOX2 shows decreased expression in endothelial cells of AD brains. MEOX2 may therefore be related to the impairment of brain vasculature in the case of AD. It is interesting to note that AD brain endothelial cells display the decreased angiogenesis capacity; nevertheless, this state can be inverted when MEOX2 is restored [254]. Endothelium cells in AD subjects have a down-regulated expression of lipoprotein receptor-related protein (LRP), a receptor involved in Aβ clearance. MEOX2-deficient mice have a down-regulation of LRP, a 50% decrease of cortical cerebral blood flow, and reduced angiogenic capability, suggesting a slower capability to clear Aβ [254]. However, it is unclear whether reduced MEOX2 expression represents a genetic risk factor for AD or is caused by the neurodegeneration processes [255, 256].

On the other hand, although vascular hypothesis provides important insights into the disease, it also has several limitations. The exact relationship between vascular abnormalities and AD is not fully understood, and it is ambiguous whether vascular changes precede or result from the pathological processes associated with AD. Even though vascular factors are believed to contribute to AD risk, the precise cause-and-effect relationship remains complex and requires further investigation [256]. Moreover, AD is characterized by the presence of Aβ plaques and neurofibrillary tangles. It is often challenging to distinguish between vascular pathology and these hallmark AD lesions. The coexistence of vascular changes and Aβ plaques in the brain complicates the interpretation of their individual contributions to the disease.

Additionally, vascular risk factors, such as hypertension, diabetes, and cardiovascular disease, are also associated with an increased risk of AD. However, these risk factors are common in the general population, and not all individuals with vascular risk factors develop into AD. Studies investigating the vascular hypothesis have reported inconsistent findings, with some demonstrating an association between vascular factors and AD, while others show conflicting results [257-260]. Since the vascular hypothesis highlights the potential importance of managing vascular risk factors for the prevention of AD, current treatments targeting vascular factors have not demonstrated significant efficacy in modifying the course of the disease [261], suggesting that additional factors beyond vascular changes may drive the pathogenesis of AD.

## 9. The intersection and interaction of key mechanisms between hypotheses

Over the years, multiple hypotheses have been proposed to understand the complex mechanisms underlying AD. The major hypotheses mentioned above are not mutually exclusive; instead, they likely interact and influence one another in a complex network of events that culminate in AD.

The amyloid cascade hypothesis highlights the role of Aβ plaques in AD pathogenesis, while the Tau hyperphosphorylation hypothesis emphasizes the formation of neurofibrillary tangles. Both hypotheses are interconnected and mutually reinforcing. Aβ accumulation can induce Tau hyperphosphorylation, thus leading to the formation of neurofibrillary tangles. In turn, Tau pathology can exacerbate Aβ deposition by impairing the clearance mechanisms of Aβ. This interplay between Aβ and Tau pathology creates a synergistic effect, thereby promoting neurodegeneration and cognitive decline [262].

The cholinergic hypothesis proposes that the degeneration of cholinergic neurons and decreased acetylcholine levels contribute to AD. Cholinergic deficits can be influenced by Aβ accumulation and neuroinflammation. Aβ plaques can directly impair cholinergic neurotransmission, while inflammation-induced damage can further exacerbate cholinergic neuronal loss. Conversely, cholinergic dysfunction may enhance Aβ deposition and promote neuroinflammation, thereby reinforcing the interplay between these hypotheses [234].

What is more, the neuroinflammation hypothesis suggests that chronic neuroinflammation plays a significant role in AD pathogenesis. Inflammation can be triggered by Aβ plaques, but it can also contribute to Aβ accumulation and Tau pathology. Inflammatory mediators released by activated microglia can promote Aβ production, impair Aβ clearance, and induce Tau hyperphosphorylation. The resulting neuroinflammation further amplifies inflammatory responses, thereby creating a self-perpetuating cycle to exacerbate neuronal damage and disease progression [263].

The oxidative stress hypothesis posits that oxidative damage contributes to AD pathology. Oxidative stress can be induced by Aβ aggregation, thus leading to ROS production and neuronal injury. In turn, oxidative stress can enhance Aβ formation and Tau phosphorylation, thus linking the oxidative stress hypothesis with the Aβ and Tau hypotheses [264]. Moreover, inflammation can exacerbate oxidative stress, further contributing to neuronal damage and dysfunction [170].

The vascular hypothesis suggests that vascular dysfunction, such as impaired cerebral blood flow and disrupted BBB integrity, contributes to AD pathogenesis. Vascular dysfunction can interact with other AD-related factors, including Aβ accumulation, Tau pathology, inflammation, and oxidative stress. Reduced cerebral blood flow can impair Aβ clearance and promote Aβ aggregation. Meanwhile, vascular dysfunction can exacerbate oxidative stress and neuroinflammation, further amplifying the cascade of events involved in AD progression [265].

Moreover, the calcium signaling hypothesis intersects with other hypotheses regarding the underlying causes of AD. For example, the amyloid cascade hypothesis proposes that the accumulation of Aβ plaques in the brain triggers a cascade of events, including the disruption of calcium homeostasis. This interaction suggests that abnormal calcium signaling may both contribute to and result from Aβ deposition [266]. In addition, dysregulated calcium signaling can contribute to Tau hyperphosphorylation, thus leading to the formation of NFTs and subsequent neuronal dysfunction [267].

Mitochondrial dysfunction hypothesis proposes that abnormalities in mitochondrial function contribute to the development and progression of AD. Emerging evidence suggests that mitochondrial dysfunction plays a significant role in AD pathogenesis and interacts with other hypotheses [268, 269]. Mitochondrial dysfunction can influence the accumulation of Aβ and Tau pathology [270]. Dysfunctional mitochondria produce increased ROS and impair the proteolytic processing of APP, thus leading to increased Aβ production [122]. In turn, Aβ can disrupt mitochondrial function by impairing mitochondrial dynamics, membrane potential, and oxidative phosphorylation. Similarly, Tau pathology can affect mitochondrial transport, dynamics, and bioenergetics, further contributing to mitochondrial dysfunction [261]. Furthermore, mitochondrial dysfunction can exacerbate cholinergic deficits in AD. Impaired mitochondrial function leads to decreased ATP production, which is crucial for synaptic transmission. Cholinergic neurons are particularly vulnerable to energy deficits and can experience energy insufficiency induced by mitochondrial dysfunction. The impaired energy supply can further impair the function of cholinergic neurons and exacerbate cholinergic dysfunction [271]. Conversely, cholinergic dysfunction can also impact mitochondrial function by reducing acetylcholine-mediated regulation of mitochondrial activities [272]. Moreover, mitochondrial dysfunction can promote inflammation and oxidative stress, while these processes, in turn, contribute to mitochondrial impairment. Dysfunctional mitochondria release pro-inflammatory molecules, activate the inflammasome, and trigger ROS production, thereby resulting in neuroinflammation and oxidative stress. Inflammation and oxidative stress can further damage mitochondria, thus leading to a vicious cycle for perpetuating AD pathology [122].

Mitochondrial and vascular dysfunctions in AD are also closely interconnected. Impaired cerebral blood flow compromises mitochondrial function by reducing oxygen and nutrient supply to neurons, which leads to energy deficits and increased mitochondrial oxidative stress. Conversely, dysfunctional mitochondria release factors that can affect vascular function, such as endothelial dysfunction and BBB disruption, further exacerbating vascular pathology in AD [273].

The mitochondrial dysfunction hypothesis appears to be a major contributor to the complex network of pathological mechanisms. Mitochondrial dysfunction can enhance Aβ and Tau pathology, cholinergic dysfunction, inflammation, oxidative stress, and vascular dysfunction. Conversely, these processes can also reciprocally influence mitochondrial function, creating a bidirectional relationship. The cumulative effect of these interactions amplifies the pathophysiological cascade, thereby contributing to the progression of AD. In a word, understanding the interplay among these hypotheses is crucial for unravelling the complex mechanisms underlying AD. A comprehensive understanding of these mechanisms will aid in the development of targeted and effective therapies for AD, ultimately improving the quality of life of millions of AD patients worldwide.

## 10. Current therapeutic approaches

Over the years, researchers have made substantial strides in understanding the underlying mechanisms of AD, thus leading to the development of various therapeutic approaches. Numerous drugs are currently used for managing AD (Table 2), including: cholinesterase inhibitors such as galantamine, donepezil, and rivastigmine, to increase the levels of acetylcholine in brain tissues for improving memory and cognitive function [274]. Memantine is a neurotransmitter used to treat moderate to severe AD, by reducing the activity of glutamate, and promoting the improvement of learning and memory capacity. Memantine blocks the activity of a specific type of glutamate receptor in the brain called the NMDA receptor that can potentially cause oxidative stress and affect the normal functioning of mitochondria. NMDA receptor antagonists can reduce excessive activity of the NMDA receptor, which is thought to contribute to neuronal damage and cognitive decline in AD [275]. Interestingly, some patients could benefit from a combination of cholinesterase inhibitors and memantine [276]. Moreover, some antidepressants, such as selective serotonin reuptake inhibitors, have been used to treat the behavioral and psychological symptoms of dementia, which are common in AD.

**Table 2:** List of common drugs used for AD.

Drug	Pros	Effectiveness	Action mechanism	Reference
Donepezil	Improving cognition and function, delaying institutionalization	Moderate	Acetylcholinesterase inhibitor	[249]
Rivastigmine	Improving cognition and function, delaying institutionalization	Moderate	Acetylcholinesterase inhibitor	[250]
Galantamine	Improving cognition and function, delaying institutionalization	Moderate	Acetylcholinesterase inhibitor and allosteric modulator of nicotinic receptors	[251]
Memantine	Improving cognition and function, delaying institutionalization	Moderate	NMDA receptor antagonist	[252]
Solanezumab	Slowing down degeneration process through increasing Aβ clearance	Limited	Remove soluble Aβ	[253]
Memantine and donepezil	Memantine plus donepezil showing superior outcomes	Limited	Combination of acetylcholinesterase inhibitor and NMDA receptor antagonist	[254]
Huperzine A	Improving cognition and function	Limited	Acetylcholinesterase inhibitor	[255]
Ginkgo biloba	Improving cognition and function	Limited	Antioxidant, anti-inflammatory, and circulatory effects	[256]
NSAIDs	No significant effect on cognition or overall AD severity.	Limited	Anti-inflammatory effect	[257]
Statins	May reduce risk of Alzheimer's disease	Limited	Cholesterol-lowering effect	[258]
Antidepressants	Improving mood and reducing behavioral symptoms	Limited	Various mechanisms	[259]

Although some studies have shown that anti-inflammatory drugs including NSAIDs such as diclofenac are used to treat AD are notably linked to reducing the incidence of AD and slowing the progression of cognitive function decline [277], NSAIDs are selective cyclooxygenase inhibitors for merely reducing the prostaglandins generation without affecting other proinflammatory mediators, and have limited anti-inflammatory activity. On the other hand, cytokine-suppressive anti-inflammatory drugs have a greater spectrum of activities since they can reduce the synthesis of pro-inflammatory cytokines, such as IL-1, TNF-α, and IL-6 [278, 279]. Therefore, it is proposed that cytokine-suppressive anti-inflammatory drugs have a greater spectrum of anti-inflammatory actions than traditional NSAIDs and could possess beneficial effects against chronic neuroinflammation [280].

Aducanumab and gantenerumab are favorably target the insoluble type of Aβ fibrils and plaques more than Aβ plaques, while lecanemab (BAN2401) selectively targets Aβ oligomers [281-283]. However, aducanumab and gantenerumab have not shown significant efficacy during the treatment of AD.

Various drugs that target mitochondrial function and oxidative stress have also been used. Donepezil has antioxidant properties for protecting mitochondria from damage [284]. Resveratrol, a natural compound found in grapes, berries, and other plants, has been shown to have anti-inflammatory and antioxidant properties and to improve mitochondrial function, in addition to inducing autophagy [285, 286]. Moreover, coenzyme Q10 has been used to improve mitochondrial function and reduce oxidative stress in cells. Some *in vivo* studies have suggested that coenzyme Q10 may have potential as a treatment for AD, however further clinical studies are needed [287]. However, most discovered drugs have not shown significant improvement, especially in phase III clinical trials [288].

On the other hand, there are some non-drug therapeutical strategies, such as cognitive stimulation therapy and reality orientation therapy, with a focus on improving cognitive function and quality of life [289, 290]. Lifestyle changes such as exercise, social engagement, and cognitive training can also be beneficial [291, 292]. It is critical to note that these approaches can be helpful for managing the symptoms of AD, but there is currently no cure for this condition. The Food and Drug Administration (FDA) in the USA has authorized only several drugs to treat disease symptoms. Cholinesterase inhibitors, such as Razadyne (galantamine®), Aricept (donepezil®), and Exelon (rivastigmine®), are cholinesterase inhibitors to increase the acetylcholine level for the involvement of judgment, memory, and other thought processes. Another drug, Namenda (memantine®), controls glutamate activity and plays a role in processing, storing, and retrieving information. A novel fixed-dose capsule containing memantine and donepezil is finally approved as Namzaric [293]. Moreover, the application of iPSC-differentiated neurons has emerged as a promising technique for further understanding of AD. Recent methods for differentiating iPSCs into cerebral organoids, glial cells, and neural stem cells have been established. However, future studies are necessary to increase the maturity and the purity of obtained cells, as well as standardize differentiation processes to ensure reproducible outcomes [294]. Although animal models, such as transgenic mice, have been used for a vast bulk of therapeutic and mechanistic studies in the field of AD, successful preclinical trials of AD treatments have failed to demonstrate clinical benefits in patients with AD, which could be attributed to the fact that the pathogenic phenotype of AD in transgenic mice does not accurately reflect human AD pathology. Additionally, regional brain atrophy and extensive neurodegeneration, which are characteristics of AD, are absent in AD transgenic mice [295].

## 11. Paving the way for effective therapeutic strategies, prevention, and cure of AD

Developing effective therapeutic strategies or finding the eventual prevention or cure for AD requires a comprehensive approach that considers various pathogenesis hypotheses. Although there is still much research to be conducted, the focuses on several potential strategies and areas have emerged. The amyloid cascade hypothesis suggests that the accumulation of Aβ plaques in the brain is a central event in AD pathogenesis. Therefore, current therapeutic approaches aim to prevent Aβ production or promote Aβ clearance, including the applications of β-secretase and γ-secretase inhibitors and immunotherapy targeting Aβ [296]. Regarding Tau protein abnormalities, irregular phosphorylation and aggregation of Tau protein are associated with NFTs, another hallmark of AD. Therefore, the development of treatments for targeting Tau pathology, such as Tau aggregation inhibitors and anti-Tau antibodies, may hold promise in slowing or preventing disease progression [297].

Based on the inflammation hypothesis, anti-inflammatory drugs and immunomodulatory therapies should be explored to reduce inflammation and regulate immune responses in the brain either by inhibiting pro-inflammatory cytokines or promoting the production of anti-inflammatory cytokines [298, 299]. Furthermore, microglial activation could also be a treatment target to promote the clearance of Aβ and Tau for minimizing detrimental inflammatory responses [300].

Regarding the cholinergic hypothesis, dysfunction in the cholinergic system contributes to cognitive decline in AD. Cholinesterase inhibitors that increase acetylcholine availability are commonly used to manage symptoms, and there are an increasing number of studies that aim to develop more effective cholinergic therapies [238]. Moreover, managing vascular risk factors, such as hypertension and diabetes, and optimizing vascular health through lifestyle modifications and medication may be helpful to reduce the impact of vascular factors on AD progression.

On the other hand, developing therapies that protect and promote the survival of neurons and synapses is a promising avenue, including therapies using neurotrophic factors, antioxidants, and agents that can promote neuroplasticity and synaptic function.

Since AD is a complex and heterogeneous disease, the development of individually-tailored therapies based on individual characteristics, genetic profiles, and disease stage may improve treatment outcomes. Precision medicine approaches, including biomarker-based diagnosis and targeted therapies, are being explored. Furthermore, combinatorial therapies that target multiple signal pathways simultaneously or sequentially could be a promising approach. For example, the combination of drugs targeting Aβ, Tau, inflammation and neuroprotection could offer synergistic effects and increase therapeutic efficacy [301].

It is crucial to highlight that early diagnosis and intervention before the occurrence of significant brain damage are important. The development of reliable biomarkers and non-invasive imaging techniques to evaluate AD pathology at its early stages will facilitate timely interventions. The lifestyle modifications including healthy diets, regular exercise, cognitive stimulation, and social engagement have been shown to have a positive impact on brain health [302]. Overall, the development of effective therapeutic strategies and the eventual prevention or cure of AD requires a comprehensive understanding of the underlying mechanisms for AD pathogenesis and a multi-faceted approach targeting its various contributing factors.

## 12. Conclusion

AD remains a significant global health issue, accounting for a large proportion of all dementia cases. The complexity of the underlying mechanisms and the interplay between various pathological factors necessitate a comprehensive understanding of this disease. This article has provided a thorough study on the major hypotheses of AD pathogenesis, with highlights of strengths, weaknesses, and potential interconnections. Although the exact cause or precise mechanism of AD is not fully understood, recent studies have made significant strides in elucidating the underlying molecular mechanisms of this disease. With a more nuanced understanding of the complex interplay among different factors that contribute to AD pathogenesis, more targeted approaches for the prevention and treatment of this disease, such as personalized medication approaches, have been developed, and new targets for AD treatments have also been identified.
